# Node-Structured Integrative Gaussian Graphical Model Guided by Pathway Information

**DOI:** 10.1155/2017/8520480

**Published:** 2017-04-12

**Authors:** SungHwan Kim, Jae-Hwan Jhong, JungJun Lee, Ja-Yong Koo, ByungYong Lee, SungWon Han

**Affiliations:** ^1^Department of Statistics, Keimyung University, Daegu, Republic of Korea; ^2^The Institute of Natural Science, Keimyung University, Daegu, Republic of Korea; ^3^Department of Statistics, Korea University, Seoul, Republic of Korea; ^4^Graduate School of Information Security, Korea University, Seoul, Republic of Korea; ^5^School of Industrial Management Engineering, Korea University, Seoul, Republic of Korea

## Abstract

Up to date, many biological pathways related to cancer have been extensively applied thanks to outputs of burgeoning biomedical research. This leads to a new technical challenge of exploring and validating biological pathways that can characterize transcriptomic mechanisms across different disease subtypes. In pursuit of accommodating multiple studies, the joint Gaussian graphical model was previously proposed to incorporate nonzero edge effects. However, this model is inevitably dependent on post hoc analysis in order to confirm biological significance. To circumvent this drawback, we attempt not only to combine transcriptomic data but also to embed pathway information, well-ascertained biological evidence as such, into the model. To this end, we propose a novel statistical framework for fitting joint Gaussian graphical model simultaneously with informative pathways consistently expressed across multiple studies. In theory, structured nodes can be prespecified with multiple genes. The optimization rule employs the structured input-output lasso model, in order to estimate a sparse precision matrix constructed by simultaneous effects of multiple studies and structured nodes. With an application to breast cancer data sets, we found that the proposed model is superior in efficiently capturing structures of biological evidence (e.g., pathways). An R software package nsiGGM is publicly available at author's webpage.

## 1. Introduction

Genomic data have been extensively applied to analyze disease mechanism on the basis of predictive signatures from DNA alterations (e.g., genotyping and mutation), RNA transcription (e.g., gene or isoform expression and fusion transcripts), and gene regulation by epigenetic changes (e.g., methylation, protein-DNA interaction, and miRNA expression). In particular, gene regulation is a complicated system that builds on tens of thousands of cellular components' interactions and diverse activities across multiple layers. Biological networks are the most popularly used data resource to sketch this interconnectivity of gene regulations. High-throughput genomic technologies are paving the way toward systematically characterizing diverse types of biological networks and suggestive of underlying gene regulation mechanisms. And yet a complete inference of network's complexity has been a long concern in the field of systems biology.

To circumvent the shortcoming of single feature-based analysis, the activity of a gene or of a whole biological process in a disease can be assessed by sets of genes (a.k.a. gene set enrichment analysis or pathway analysis). In doing so, a bulk of pathways have been identified through many cancer-related researches [1]. Pathway information demonstrates cellular functions and biological processes or represents a unique signature of deregulation of a given gene [2]. For example, the pathway or signature associated with the activity of a given oncogene is defined as the set composed of those genes most differentially expressed by perturbation of oncogenes [3–5]. Importantly, the usage of pathway information is increasingly prevalent in biomedicine. For instance, target drug associated with potential pathway is taken as a practical solution to overcome the traditional drug discovery that usually adopts the one-drug-one-target approach. This strategy takes into account the fact that the disease occurrence is usually the result of complex interactions of molecular events.

In recent years, large-scale genomic data generated from relevant biological experiments or clinical hypotheses have increasingly soared, as high-throughput experiment technologies have markedly advanced [6]. Such increasing genomic data has been publicly available in data repositories (e.g., Gene Expression Omnibus and Sequence Read Archive). This abundance of biological experiments poses a new challenge of multiple data in regard to exploring and validating biological signatures and pathways. More precisely, a question of network analysis often relates to how to characterize underlying transcriptomic patterns or molecular mechanisms across disease subtypes or between case-control groups, because it is commonplace that biological signals are not coherently present across studies. Generally a single network [7–9] is found to accurately estimate underlying dependency with an adjustment of gene perturbation effects (e.g., polymorphic genotype alteration [10, 11]). Nonetheless, these methods hardly discover network patterns of subtle signals and dynamic features in the midst of coupled networks under diverse conditions. Moreover, single networks potentially generate many potential false positive signals (edges) attributed to experimental biases and errors. To address this challenge, the recent trend of data analysis has been in the spotlight to data integration allowing for multiple data to achieve a more accurate network inference. To this end, many have proposed methods to combine multiple networks based on unified model [12–14]. This approach is also known as integrative analysis and is analogue to traditional meta-analysis.

The joint Gaussian graphical model (JGGM; Danaher et al. [12]) focuses on incorporating nonzero edge effects (i.e., off-diagonal entries of precision matrix) to combine multiple studies in view of integrative analysis. This model, however, inevitably is dependent on post hoc analysis when validating biological significance. Therefore it is interesting to combine not only DNA and/or transcriptomic changes but also pathway information as such well-ascertained biological evidence. Normally we perform post hoc analysis to see if the estimated gene networks are enriched for any pathways. Contrary to this, it is also sensible to estimate gene networks, with an adjustment of pathway information. It is common that we hardly combine pathway information in spite of its biological significance. To the best of our knowledge, no method has been proposed that can accommodate overlapping node structures, mainly due to overlapped gene annotations of pathway gene sets. To tackle this problem, we propose a new graphical model called “node-structured integrative Gaussian graphical model (nsiGGM)” jointly leveraging a priori knowledge of pathway information. This method allows for overlapping group lasso problems, making it possible to integrate overlapped genes of pathways. It is worthwhile for biological pathways to intervene the network estimation to reveal true gene regulatory network. The nsiGGM builds on prespecified structured nodes with multiple genes as building blocks in the stage of estimating a precision matrix. The implementation rule employs *l*_1_/*l*_2_ lasso penalty of structured input-output lasso model [15], in order to estimate sparse precision matrix that accounts for simultaneous effects of multiple studies and structured nodes. With an application to simulated and breast cancer genomic data, the proposed model is found to be superior in efficiently capturing transcriptional modules predefined by pathway database. A software package (nsiGGM) is publicly available at author's webpage (https://sites.google.com/site/sunghwanshome/).

This paper is outlined as follows. In Section 2, we review background knowledge of the standard and joint Gaussian graphical models. In addition, we propose the node-structured integrative Gaussian graphical model (nsiGGM). In Section 3, we describe an implementation strategy that is primarily based on the input-output lasso. In Section 4, we compare performance of our proposed methods with other methods using real breast cancer data (TCGA) and simulated data. In Section 5, conclusions and further studies are discussed.

## 2. Method

In this section, we briefly discuss methodological backgrounds on the Gaussian graphical models (GGM) aiming at constructing gene networks. In what follows, we propose the node-structured integrative Gaussian graphical model (nsiGGM) that can accommodate a priori biological knowledge (e.g., pathway data or targeted predictive genes of miRNA).

### 2.1. Gaussian Graphical Models for Gene Networks

A Gaussian graphical model demonstrates the conditional dependency of multiple random variables, *Y*_1_,…, *Y*_*p*_, with a graph *G* = (*V*, *E*), where *V* = {1,…, *p*} is a set of nodes and *E* is a set of edges indicating that nodes are linked and conditionally dependent. Let *Y* follow the multivariate Gaussian distribution *N*_*p*_(0, Σ), where Σ is a *p* × *p* covariance matrix. Let Σ^−1^ = Θ denote the inverse covariance matrix (also known as a precision matrix). More precisely, each nonzero off-diagonal element *θ*_*ij*_ implies conditional dependency between the *i*th and *j*th nodes given all the other variables, *i*, *j* = 1,…, *p*, whereas the covariance Σ presents marginal dependencies without considering other variables. This model is also called a GGM [16]. The graphical lasso [9, 17] produces a sparse Gaussian graphical model constructed in nonpenalized edges in Θ. The graphical lasso minimizes the negative log-likelihood with the *L*_1_ lasso penalty:

(1) $$\begin{array}{c} \underset{\Theta }{arg\;min}-\log det\;\Theta +tr\left(\;S\Theta \;\right)+\lambda {\left\|\;\Theta \;\right\|}_{1}, \end{array}$$

where tr(*A*) is the trace of matrix *A*, *S* is the sample covariance matrix, and ‖Θ‖_1_ = ∑_*i*_∑_*j*_|*θ*_*ij*_| is the regularization parameter adjusting the degree of sparsity. The optimal value for *λ* can be chosen by cross-validation or the Bayesian information criterion (BIC; Schwarz [18]; Yuan and Lin [8]).

### 2.2. Joint Gaussian Graphical Models for Combining Multiple Studies

In this section, we revisit the joint Gaussian graphical models (JGGM) proposed by Danaher et al. [12]. Simply put, the JGGM combines multiple studies and constructs multiple networks in a unified model. Let *K* denote the number of studies in our data and {Σ^−1^} = (Σ_1_^−1^,…, Σ_*K*_^−1^) the true precision matrices. Consider genomic data of *K* studies, *Y*^(1)^,…, *Y*^(*K*)^, each of which consists of *n*_*k*_ samples with *p* common features, where *K* ≥ 2. We assume that ∑_*k*=1_^*K*^*n*_*k*_ observations are independent and that those of each data set follow the multivariate normal distribution as *Y*^(*k*)^ ~ *N*_*p*_(*μ*_*k*_, Σ_*k*_) for 1 ≤ *k* ≤ *K*. It is well known in meta-analysis that multiple data sets are of common associations and genomic characteristics among features (e.g., genetic association intensity). It, therefore, is worth estimating precision matrices across *K* studies in parallel rather than separate estimation. To this end, we assume that the features within each data set are centered and take the form of a penalized log-likelihood with the group sparsity-inducing *L*_2_ penalty that maximizes (2) with respect to {Θ}:

(2) ∑k=1Knklog⁡det⁡Θk−trSkΘk+λ1∑k=1K ∑i≠jθijk+λ2∑i≠j∑k=1Kθijk21/2

subject to {Θ} = (Θ^(1)^,…, Θ^(*K*)^) being positive definite, where *S*^(*k*)^ = (1/*n*_*k*_)(*Y*^(*k*)^)^*T*^*Y*^(*k*)^ is the sample covariance matrix of *Y*^(*k*)^ and *λ*_1_, *λ*_2_ are nonnegative tuning parameters. It is interesting to note that the *L*_2_-penalty captures similarity across the *K* precision matrices. Due to this property, the penalty terms of (2) are also referred to as the joint graphical lasso (JGL). Moreover, the *L*_1_ penalty induces estimated precision matrices to be sparse.

### 2.3. Node-Structured Integrative Gaussian Graphical Model

In this section, we propose an integrative graphical model that can accommodate a priori known structure of genomic features. Learning gene networks, the sparseness of precision matrix can be guided to some extent by known feature modules (e.g., pathway information). Typically data integration allows picturing the interplay of underlying biological factors. In this regard, it is worthwhile accommodating known feature module information ascertained in previous experiments. In doing so, we seek to integrate a priori feature module information to be embedded across multiple networks via an additional *L*_2_ group penalty. The following objective function is taken to minimize

(3) ∑k=1Knklog⁡detΘk−trSkΘk+λ1∑k=1K ∑i≠jθijk+λ2∑i≠j∑k=1Kθijk21/2+λ3∑k=1K ∑gm∈GΘgmk2,

where **g**_*m*_ is a subset of off-diagonal entry indices of Θ for 0 ≤ *m* ≤ *M*, *G* = {**g**_1_,…, **g**_*M*_}, *M* is the number of a priori feature modules, and ‖Θ_**g**_*m*__^(*k*)^‖_2_ = (∑_(*i*,*j*)∈**g**_*m*__^ ^*θ*_*ij*_^(*k*)^^2^)^1/2^. Importantly, it is noted that elements of **g**_*m*_ can be overlapped (e.g., duplicated genes of two different pathways). The third penalty, ‖Θ_**g**_*m*__^(*k*)^‖_2_ adjusted by *λ*_3_ ≥ 0 pertains to structured feature modules (i.e., structured node in networks) on the basis of a priori known information. Here, unbiased regularization to each feature should be taken into consideration, in the sense that the feature overlapping inevitably comes into play.

In what follows, we present a toy example to demonstrate how a priori information constructs feature modules in Θ_**g**_*m*__^(*k*)^. In Figure S1, in Supplementary Material available online at https://doi.org/10.1155/2017/8520480, we take an example of networks consisting of 5 common nodes (e.g., genomic features) across three studies. In Figure S1A, the second penalty with *λ*_2_ captures matched up common edges (e.g., *θ*_14_^(1)^, *θ*_14_^(2)^, *θ*_14_^(3)^) identical to the joint graphical lasso. Besides, the third group lasso penalty with *λ*_3_ accommodates the six edges of the three features in a predefined module Θ_**g**_1__^(*k*)^ so that feature regulatory effects can be further modeled in the context of data integration (see Figure S1B). Importantly note that this module structure (e.g., pathway) is priorly known knowledge. It is interesting that this approach is in line with the integrative cluster [19] that allows for* cis*-regulatory effects and target gene prediction for miRNAs. In the case of multiple modules in network, suppose that we are given a set of five genes {*X*_*i*_} and a precision matrix {*θ*_*ij*_} for 1 ≤ *i*, *j* ≤ 5. Let a priori information generate two feature modules defined as Module 1, {*X*_1_, *X*_2_, *X*_3_, *X*_4_}, and Module 2, {*X*_3_, *X*_4_, *X*_5_}, and then we can enumerate precision matrix's index (*i*, *j*) of each module for all *i*, *j*, say, **g**_1_ = {(1,2), (1,3), (1,4), (2,3), (2,4), (3,4)} and **g**_2_ = {(3,4), (3,5), (4,5)}. Of note, the component (3,4) is simultaneously present in both **g**_1_ and **g**_2_, implicating that a suitable implementation is required for regularization to the overlapped component (3,4). To estimate solutions to (3), we apply the structured input-output lasso [15] that can handle overlapped features, making it possible to learn a model allowing for both single-node effects across studies and predefined node structures (e.g., pathway modules). Inspired by integrative nature of this method, we call this graphical model the node-structured integrative Gaussian graphical model (nsiGGM). When it comes to tuning the penalty parameters (*λ*_1_, *λ*_2_, and *λ*_3_), the BIC is applied to determine the optimal sparseness of networks' edges.

## 3. Implementation Strategy

### 3.1. Structured Alternating Directions Method of Multipliers Algorithm

In this section, we delineate the implementation strategy for the nsiGGM. We solve problem (3) by using structured alternating directions method of multipliers algorithm (sADMM). The alternating directions method of multipliers algorithm (ADMM) was previously introduced to tackle the problem of the JGL [12]. Similar to the JGL, the sADMM proposed in spirit of the ADMM is designed to adopt the structured input-output lasso in order to embed node structures into the model. We first reformulate (3) with *P*(Θ) and *Z* as

(4) maximizeΘ,Z −∑k=1Knklog⁡det⁡Θk−trSkΘk+PZ,

where *P*({*Z*}) = *λ*_1_∑_*k*=1_^*K*^∑_*i*≠*j*_|*Z*_*ij*_^(*k*)^| + *λ*_2_∑_*i*≠*j*_(∑_*k*=1_^*K*^*Z*_*ij*_^(*k*)^^2^)^1/2^ + *λ*_3_∑_*k*=1_^*K*^∑_**g**_*m*_∈*G*_‖*Z*_**g**_*m*__^(*k*)^‖_2_; *Z*^(*k*)^ = Θ^(*k*)^ for 1 ≤ *k* ≤ *K* and {*Z*} = (*Z*^(1)^,…, *Z*^(*K*)^) that satisfies positive definiteness. Boyd et al. [20] proposed the scaled augmented Lagrangian to solve problem (4) by

(5) LΘ,Z,U=−∑k=1Knklog⁡det⁡Θk−trSkΘk+PZ+12∑k=1KΘk−Zk+UkF2−12∑k=1KUkF2,

where {*U*} = (*U*^(1)^,…, *U*^(*K*)^) are dual variables and ‖*A*‖_*F*_ denotes the Frobenius norm of matrix *A* (i.e., ${\left\|A\right\|}_{F}=\sqrt{\sum_{i}\sum_{j}A_{ij}^{2}}$). The sADMM algorithm repeatedly solves the three-step optimization with respect to Θ_(*i*)_, *Z*_(*i*)_, and *U*_(*i*)_, starting with initial values of the related parameters: Θ^(*k*)^ = *I*, *U*^(*k*)^ = 0, and *Z*^(*k*)^ = 0 for 1 ≤ *k* ≤ *K*. The iteration is repeated until convergence as follows: In Θ-step for 1 ≤ *k* ≤ *K*, update Θ^(*k*)^ that minimizes

(6) −∑k=1Knklog⁡det⁡Θk−trSkΘk+12∑k=1KΘk−Zi−1k+Ui−1kF2.

In *Z*-step, for *k* = 1,…, *K*, update *Z*^(*k*)^ that minimizes

(7) 12∑k=1KZk−AikF2+λ1∑k=1K ∑i≠jZijk+λ2∑i≠j∑k=1KZijk21/2+λ3∑k=1K ∑gm∈GZgmk2,

where *A*_(*i*)_^(*k*)^ = Θ_(*i*)_^(*k*)^ + *U*_(*i*−1)_^(*k*)^. To find the optimal solution of (7), we directly apply the structured input-output lasso [15] to (7) using both coordinate descent algorithm and KKT conditions considered to boost up the computational speed. For more details, see [15]. In *U*-step, for *k* = 1,…, *K*, update *U*^(*k*)^ as *U*_(*i*−1)_^(*k*)^ + Θ_(*i*)_^(*k*)^ − *Z*_(*i*)_^(*k*)^. Update repeatedly the three parameters until convergence by a stopping rule below:

(8) $$\begin{array}{c} \frac{\sum_{k}{\left\|\Theta_{\left(i\right)}^{\left(k\right)}-\Theta_{\left(i-1\right)}^{\left(k\right)}\right\|}_{1}}{\sum_{k}{\left\|\Theta_{\left(i-1\right)}^{\left(k\right)}\right\|}_{1}}<10^{-3}. \end{array}$$

Putting together, Algorithm 1 encapsulates the structured alternating directions method of multipliers algorithm.

## 4. Numerical Studies

### 4.1. Simulated Data

In this section, we carry out experimental studies to assess performance of the nsiGGM. In brief, the following describes how we generate simulated data. The experimental scheme is largely motivated by Chun et al. [14]. Let *K* be the total number of studies, each containing true signal genes *p* = 40 for a priori module (e.g., pathway genes) and sample size *n*^(*k*)^ = 100, where 1 ≤ *k* ≤ *K* (=3). Starting off with edges of signal genes, we first generate network edges of 100 nodes subject to the scale-free network structures, the most commonly observed structures in biology, being simulated by applying the Barabasi Albert algorithm [21]. Subsequent to this, we randomly added four edges to impose random effects. Constructing network structures, we simulate the precision matrices by setting values of the off-diagonals sampled from Unif(−0.1,0.1) and by setting the diagonal elements with ∑_*j*≠*i*_|*θ*_*i*,*j*_^(*k*)^|. The process is repeated until Θ^(*k*)^ becomes a positive definite matrix. For simulating *Y*^(*k*)^, we first consider a scenario such that no covariate incurs dependency among genes. Thus, this is an ideal experiment scenario in that any conditional dependency is not taken into consideration to the model. We simulated *Y*^(*k*)^, where each *i*th row of *Y*^(*k*)^ was randomly sampled from *N*_*p*_(0, Θ^(*k*)^−1^^). Simulations were repeated and average values are presented in Tables 1 and 2. To examine performance of the nsiGGM, sensitivity, specificity, and Youden index were benchmarked by comparing the JGGM [12] and GGM [16]. Youden index is defined as Sensitivity + Specificity − 1, ranging from −1 to 1. In principle, the higher the Youden index, the higher the prediction accuracy.

In Table 1, Youden index of the nsiGGM appears to be clearly declining as noise edges increase in number and yet is consistently larger than that shown in the JGGM and GGM. This is mainly due to the fact that the JGGM suffers low specificity (0.8685–0.8733) compared to the nsiGGM (0.9433–0.9481). In contrast, the JGGM slightly outperforms, when 30 and 40 noises are augmented, the nsiGGM for sensitivity at the expense of poor specificity. Taken together, it is clear to say that the nsiGGM is superior to the JGGM and GGM in detecting the true underlying pathway sets.

### 4.2. Application to Genomic Data

**Figure 1:**
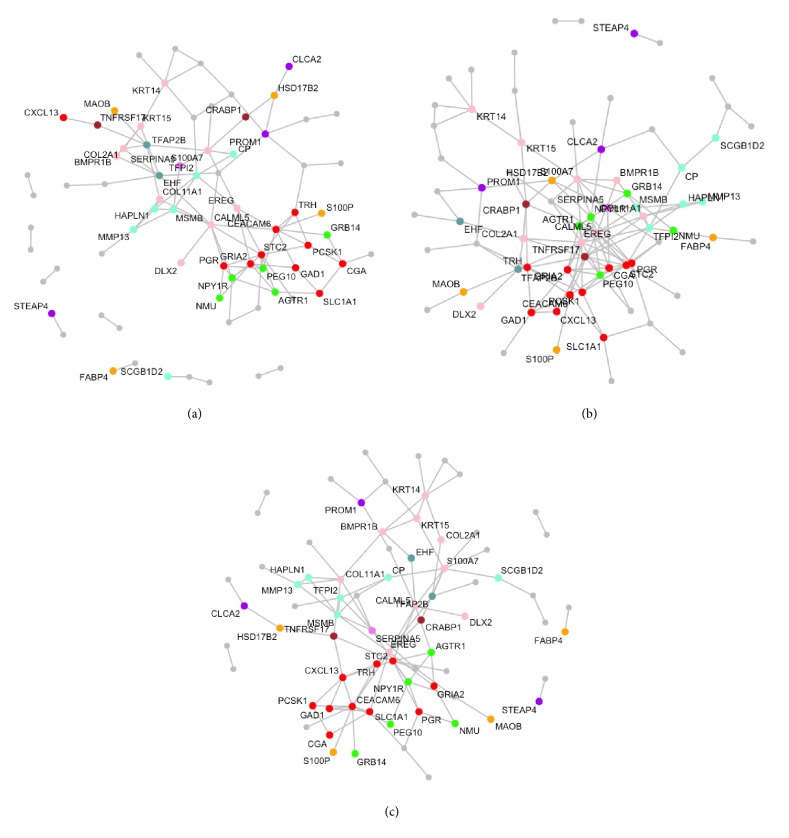
Three gene networks estimated by the nsiGGM. The detection rate of pathway genes is 0.573.

**Figure 2:**
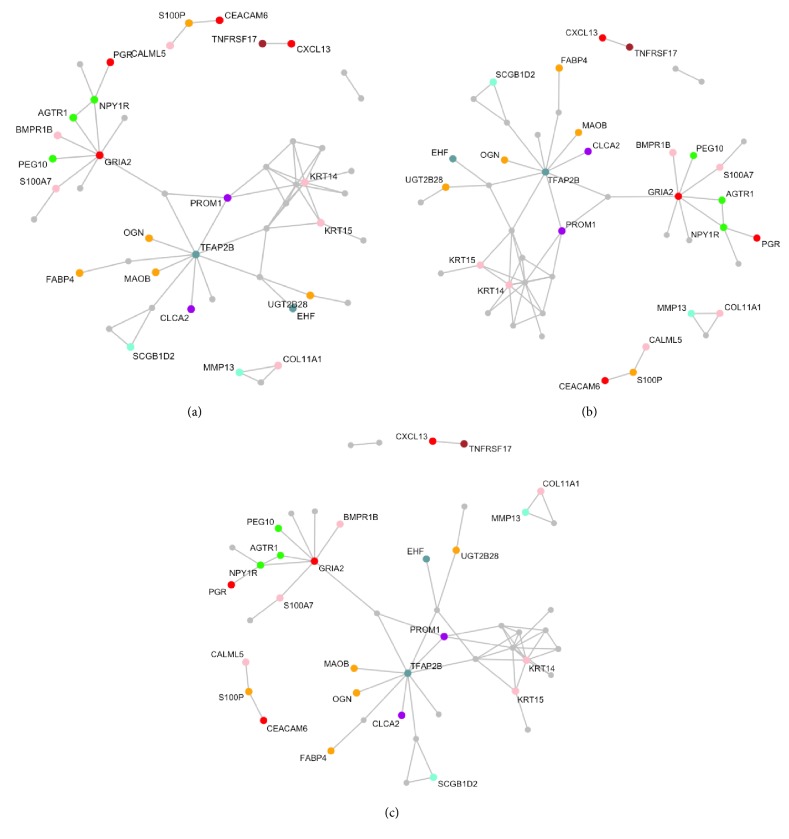
Three gene networks estimated by the JGGM. The detection rate of pathway genes is 0.521.

In this section, we demonstrate applications to three mRNA expression profiles for breast cancer. We collected two microarray profiles from Desmedt et al. [22], Wang et al. [23], and TCGA cancers data from TCGA's web portal (https://cancergenome.nih.gov/), where we retrieved mRNA data of breast carcinoma (BRCA). We matched up features across all studies and filtered out probes by the rank sum of mean and standard deviation (SD < 0.99; Wang et al. [24]), which selected 106 genes.  Table 2 delineates detailed information of miRNA expression data. In what follows, we examine if the nsiGGM is suited to improve accuracy for detecting pathway genes. We collected gene sets from exploring the Molecular Signatures Database (MSigDB) v2.5 gene set collections [25], consisting of at least 11 genes of 106 genes, of which 53 distinct genes belong to the 11 pathways presented in Table 3. To evaluate a detection rate of pathway genes, we define an evaluation benchmark, *R*(·) as follows:

(9) $$\begin{array}{c} R\left(\;\Phi_{j}\;\right)=\frac{\sum_{i\in \Phi_{j}}I\left(ith\;\;gene\;\;of\;\;jth\;\;network\text{'}s\;\;node\;\;belongs\;\;to\;\;any\;\;of\;\;given\;\;pathways\right)}{\#\;\;of\;\;total\;\;selecte\;\;dnodes}, \end{array}$$

where Φ_*j*_ is a set of gene indices, whose genes form *j*th network and *I*(·) is an indicator function. Comparing the JGGM, we examine whether the nsiGGM effectively captures the existing pathway structures better than the JGGM in context of connectivity and proportions of identified pathway genes. To first appearances, the nsiGGM effectively represents modules well enriched with pathway genes in Figure 1, as compared to those of the JGGM in Figure 2. In support of this notion, given that we observed ∑_*j*_*R*(Φ_*j*_) of nsiGGM = 0.573 and ∑_*j*_*R*(Φ_*j*_) of JGGM = 0.521, where 1 ≤ *j* ≤ 3, it is not surprising to say that the nsiGGM can facilitate constructing gene networks biologically more enriched for pathway gene sets than the JGGM. Table 3 enumerates the pathway genes discovered by the nsiGGM, each being highlighted by bold and underlined characters (note: asterisks represent pathway genes identified by only the nsiGGM not by JGGM). Interestingly, there are many pathways genes monitored by the nsiGGM, but not by the JGGM. Focusing on the cell signaling pathway, we particularly notice that EREG [26], SLC1A1 [27], STC2 [28], GAD1 [29], and TRH [30] are genes not selected by the JGGM but nonetheless previously were monitored in signaling pathways. Importantly, Hou et al. [28] showed that STC2 inhibited tumorigenesis and metastasis of breast cancer cells, indicating that STC2 may inhibit epithelial-mesenchymal transition (EMT) at least partially through the PKC/Claudin-1-mediated signaling in human breast cancer cells. Therefore, STC2 can be taken into consideration as a potential biomarker for metastasis and targeted therapy in human breast cancer. Besides, signaling through glutamate receptors in regard to SLC1A1 has been reported in human cancers [31]. In support of this evidence, it is also well known that increases in SLC1A1 expression subject to hypoxia-inducible factors (HIFs) possibly contributes to increased efflux of glutamate, by which glutamate transporters and receptors are regulated to activate key signal transduction pathways that promote cancer progression [27]. Therefore, it is clear to say that the nsiGGM is superior in detecting genes capable of implicating the functional process of human cancers in essence.

## 5. Conclusion and Discussion

In this article, we propose a new graphical model called node-structured integrative Gaussian graphical model (nsiGGM) jointly learning Gaussian graphical models with an emphasis of prior knowledge of pathway information. It is highlighted that this method allows us to handle overlapping group lasso problems, making it possible to integrate overlapped pathway gene sets. With applications to experimental and real data, we verified outstanding numerical performance of the nsiGGM and analytical capability of inducing biological significance related to breast cancer. And yet it might be controversial whether prior knowledge too excessively determines the network structures. Despite apprehension to overly guided network structures, a priori known information can be still acceptable in that the nsiGGM selects tuning parameters on the basis of the likelihood-based BIC.

The proposed nsiGGM is highly subject to computational complexity in nature, mainly due to the coordinate decent algorithm to tackle the sparse overlapping group lasso. Since the sparse overlapping group lasso applied here deals with both study-specific effects and prior knowledge, the optimization becomes inevitably complicated. Our current package is implemented in R and the routine flows can be further expedited via C/C++ in the future. Currently, the prior knowledge of regulatory structure is accommodated to an unidirectional graphical model. It is also interesting that we impose the prior knowledge to directional networks instead, so that the presence or absence of directional edges amid multiple features can be explicitly modeled. We leave these tasks for future research.

## Supplementary Material

Supplementary Material provides graphical examples of group lasso regulating precision matrices of multiple studies. First, the joint graphical lasso (JGL) is taken to show how to integrate multiple elements across precision matrices. Second, the structured penalty, simulatenously, regulates pathway information.

## Figures and Tables

**Algorithm 1:**
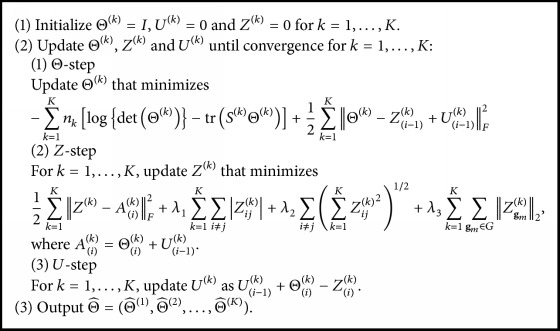
The structured alternating directions method of multipliers algorithm.

**Table 1:** Performance comparisons of the nsiGGM with the JGGM and GGM using data simulated along with predefined module genes.

Methods	# of noise genes	Sensitivity (s.e.)	Specificity (s.e.)	Youden (s.e.)
nsiGGM	30	0.2217 (0.0253)	0.9433 (0.0036)	0.1650 (0.0257)
	40	0.2125 (0.0133)	0.9472 (0.0053)	0.1598 (0.0117)
	50	0.2034 (0.019)	0.9481 (0.0035)	0.1515 (0.0175)

JGGM	30	0.2433 (0.04)	0.8685 (0.0273)	0.1118 (0.0161)
	40	0.2815 (0.0418)	0.8321 (0.0309)	0.1136 (0.0146)
	50	0.1920 (0.0425)	0.8733 (0.0318)	0.0653 (0.0124)

GGM	30	0.2593 (0.0264)	0.8325 (0.0214)	0.0918 (0.0094)
	40	0.2752 (0.029)	0.8050 (0.0257)	0.0802 (0.0074)
	50	0.2177 (0.0303)	0.8431 (0.0268)	0.0608 (0.0085)

**Table 2:** Shown are the brief descriptions of the three data information pieces used in real genomic application.

Study	Data type	# of samples	# of matched genes	Reference
Breast cancer	mRNA	319	10,676	The Cancer Genome Atlas (TCGA)
Breast cancer	mRNA	134	10,676	GSE7390
Breast cancer	mRNA	209	10,676	GSE2034

**Table 3:** The pathway sets from the Molecular Signatures Database (MSigDB) analyzed in the nsiGGM. (Note: asterisks represent pathway genes identified by only the nsiGGM not by JGGM.)

Pathway 1: extracellular region (11 genes)	
**SERPINA5**^*∗*^, **MMP13**, **EREG**^*∗*^, **HAPLN1**^*∗*^, **CP**^*∗*^, **S100A7**, CRISP3, **SCGB1D2**, **COL11A1**, **TFPI2**^*∗*^, **MSMB**^*∗*^	
Pathway 2: membrane part (11 genes)	
**EREG**^*∗*^, PTPRN2, **CLCA2**, **NPY1R**, TRPA1, **TNFRSF17**, **AGTR1**, **CEACAM6**, **SLC1A1**^*∗*^, **PROM1**, **HSD17B2**^*∗*^	
Pathway 3: membrane (14 genes)	
** EREG**^*∗*^, PTPRN2, **STEAP4**^*∗*^, **GRIA2**, **CLCA2**, **NPY1R**, TRPA1, **TNFRSF17**, **AGTR1**, **SERPINA5**^*∗*^, **CEACAM6**, **SLC1A1**^*∗*^, **PROM1**, **HSD17B2**^*∗*^	
Pathway 4: cytoplasm (13 genes)	
**OGN**, CA2, MYBPC1, NLRP2, **MAOB**, **UGT2B28**, **S100A7**, CRISP3, **PEG10**, **S100P**, **FABP4**, CLGN, **HSD17B2**^*∗*^	
Pathway 5: plasma membrane (12 genes)	
**AGTR1**, **CEACAM6**, **EREG**^*∗*^, PTPRN2, **SLC1A1**^*∗*^, **STEAP4**^*∗*^, **PROM1**, **GRIA2**, **CLCA2**, **NPY1R**, TRPA1, **TNFRSF17**	
Pathway 6: system development (12 genes)	
**EREG**^*∗*^, **TFAP2B**, **CALML5**, **KRT15**, **KRT14**, **DLX2**^*∗*^, **BMPR1B**, MSTN, **S100A7**, NKX2-2, **COL11A1**, **COL2A1**^*∗*^	
Pathway 7: signal transduction (15 genes)	
**EREG**^*∗*^, **CALML5 **, **GRIA2**, **TRH**^*∗*^, **PGR**, **NPY1R**, **CGA**^*∗*^, **CRABP1**^*∗*^, **TNFRSF17**, **AGTR1**, **CEACAM6**, **PEG10**, **GRB14**^*∗*^, **STC2**^*∗*^, **NMU**^*∗*^	
Pathway 8: multicellular organismal development (15 genes)	
**EREG**^*∗*^, **TFAP2B**, **CALML5**, **KRT15**, **KRT14**, DLX2, **BMPR1B**, MSTN, **S100A7**, NKX2-2, **COL11A1**, **EHF**, **COL2A1**^*∗*^, **CRABP1**^*∗*^, **TNFRSF17**	
Pathway 9: cell signaling (11 genes)	
**PGR**, **CEACAM6**, **EREG**^*∗*^, **PCSK1**^*∗*^, **CXCL13**, **SLC1A1**^*∗*^, **CGA**^*∗*^, **STC2**^*∗*^, **GAD1**^*∗*^, **GRIA2**, **TRH**^*∗*^	
Pathway 10: anatomical structure development (13 genes)	
**EREG**^*∗*^, **TFAP2B**, **CALML5**, **KRT15**, **KRT14**, **DLX2**^*∗*^, **BMPR1B**, MSTN, **S100A7**, NKX2-2, **COL11A1**^*∗*^, **COL2A1**, **EHF**	
Pathway 11: organ development (11 genes)	
MSTN, **EREG**^*∗*^, **CALML5**, **S100A7**, **KRT15**, **KRT14**, NKX2-2, **DLX2**^*∗*^, **COL11A1**, **COL2A1**^*∗*^, **BMPR1B**	
