# Supplementary material for: Node-Structured Integrative Gaussian Graphical Model Guided by Pathway Information
Source: Comput Math Methods Med. 2017 Apr 12;2017:8520480. doi: 10.1155/2017/8520480 (PMC5405575; doi:10.1155/2017/8520480)
Supplement: Supplementary file 1 — Supplementary Material provides graphical examples of group lasso regulating precision matrices of multiple studies. First, the joint graphical lasso (JGL) is taken to show how to integrate multiple elements across precision matrices. Second, the structured penalty, simulatenously, regulates pathway information. [file 8520480.f1.pdf]

Supplementary material: Node-structured Integrative Gaussian Graphical Model Guided by Pathway Information

SungHwan Kim[4], Jae-Hwan Jhong[1], JungJun Lee[1], Ja-Yong Koo[1], ByungYong Lee[3] and SungWon Han[2]

[1]Department of Statistics, Korea University, Seoul, South Korea

[2]School of Industrial Management Engineering, Korea University, Seoul, South Korea

[3]Graduate School of Information Security, Korea University, Seoul, South Korea

[4]Department of Statistics, Keimyung University, Daegu, South Korea

February 20, 2017

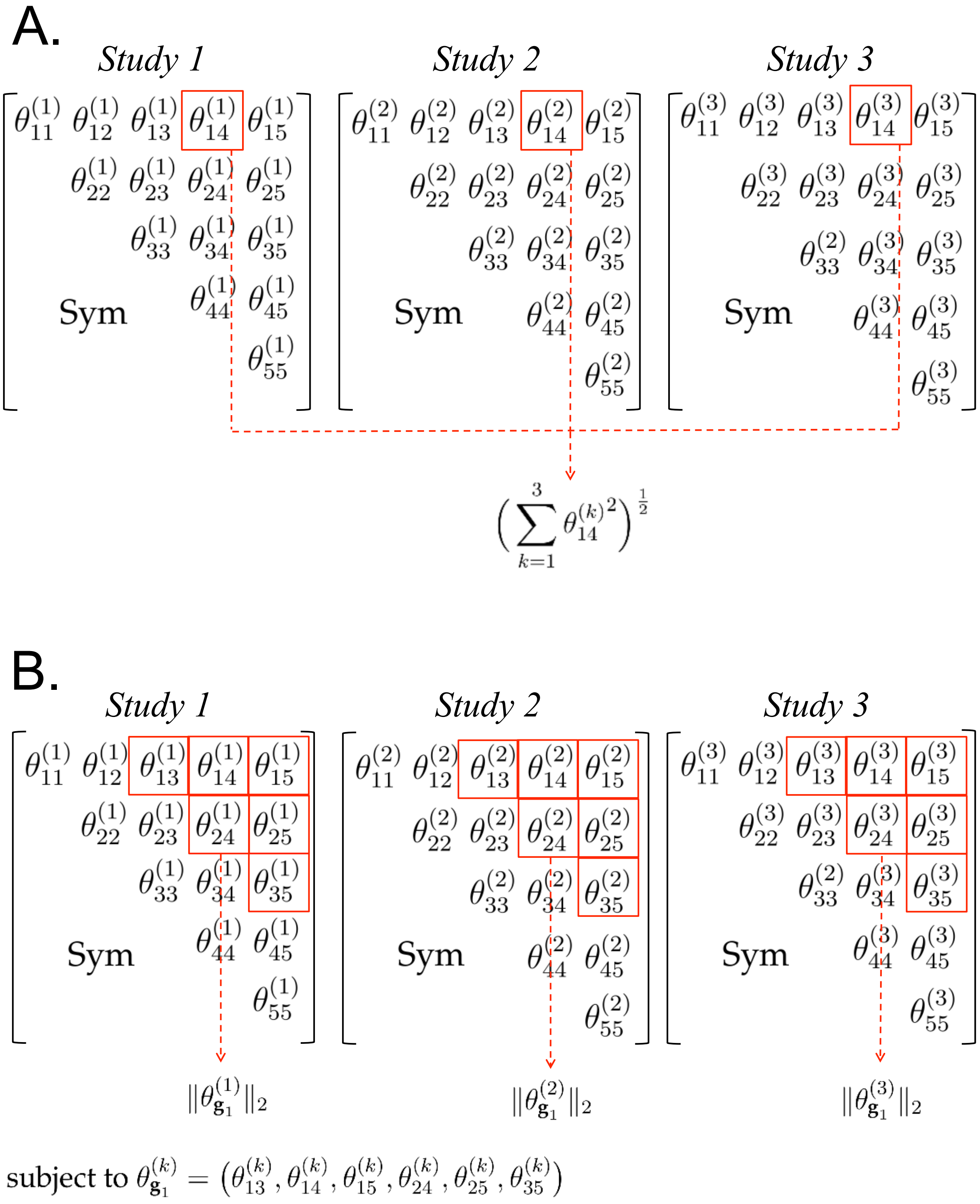

Figure 1: Shown are the examples of each group lasso regulating precision matrices of multiple studies - (A) Joint graphical lasso (JGL) integrates three elements across precision matrices introduced in Joint Gaussian graphical model (JGGL) / (B) six elements of modules g_1 that originates from three nodes in a pathway are simultaneously taken in $\|\theta_{\mathbf{g}_1}^{(k)}\|_2$ for $k = 1, 2$ and 3.
